# Comparative Readability Analysis of AI-Generated Versus Evidence-Based Educational Content on Atrial Fibrillation Management for Medical Professionals

**DOI:** 10.7759/cureus.92506

**Published:** 2025-09-17

**Authors:** Thejas Swaroop Konduru, Sharada Medapuram Belagu, Areefa Momtaz, Jasmin Maghamifar

**Affiliations:** 1 Acute Internal Medicine, Midland Metropolitan University Hospital, Sandwell and West Birmingham NHS Trust, West Midlands, GBR; 2 Respiratory Medicine, Walsall Manor Hospital, Walsall, GBR; 3 Internal Medicine, Midland Metropolitan University Hospital, Sandwell and West Birmingham NHS Trust, West Midlands, GBR

**Keywords:** artificial intelligence in healthcare, atrial fibrillation, chatgpt, evidence-based medicine, readability analysis, uptodate

## Abstract

Introduction

Atrial fibrillation (AF) is the most common sustained arrhythmia and is associated with increased risks of stroke, heart failure, and healthcare burden. Access to clear and up-to-date educational content is essential for effective decision-making in complex cases such as AF. Evidence-based resources like UpToDate are often time-consuming to read, and clinicians frequently face time constraints in fast-paced clinical settings. With the growing role of artificial intelligence in healthcare, tools like ChatGPT-3.5 (OpenAI, San Francisco, CA, USA) offer fast and accessible medical summaries. However, their suitability in professional education remains inadequately studied, particularly in comparison with evidence-based resources like UpToDate.

Methodology

A cross-sectional study was conducted in June 2025. Educational content was generated using ChatGPT-3.5 based on structured prompts and retrieved from UpToDate. Non-textual elements were excluded. Readability was assessed using the Flesch-Kincaid Reading Ease (FRE) score, the Flesch-Kincaid Grade Level (FKGL), the Simple Measure of Gobbledygook (SMOG) Index, word count, sentence count, average words per sentence, and both count and percentage of difficult words. Statistical comparison was done using the Mann-Whitney U test (p < 0.05), analysed with R software (v4.3.2; R Foundation for Statistical Computing, Vienna, Austria).

Results

ChatGPT content was significantly shorter (median 495 vs. 3381 words; p = 0.029), had shorter sentences (14.3 vs. 19.3 words; p = 0.029), but a higher percentage of difficult words (29.6% vs. 23.3%; p = 0.029). Other differences were not statistically significant.

Conclusions

ChatGPT provides concise educational content with readability scores comparable to UpToDate but with a higher proportion of complex vocabulary. While promising as a supplementary resource, its integration into clinical decision-making should be guided by expert review and validation.

## Introduction

Atrial fibrillation (AF) is the most common sustained arrhythmia globally and is associated with marked increases in morbidity, stroke risk, heart failure, and overall healthcare burden [1-3]. In recent years, the readability of educational content on AF management has become a crucial factor influencing effective clinical decision-making and knowledge transition.

Decision-making in AF management is complicated by the availability of multiple treatment options and gaps in evidence, often resulting in more than one clinically reasonable choice [3,4]. Recent guideline recommendations acknowledge this complexity and emphasize the importance of shared decision-making, which incorporates patient values, goals, and preferences into treatment decisions [3,5]. For clinicians, continuous medical education is essential to remain updated on evolving diagnostic criteria and treatment protocols. Two decades ago, general internists would have had to read approximately 17 articles per day to stay current with the primary clinical literature - a figure that has continued to rise [6].

This challenge is compounded by the limited time available in clinical practice, with clinicians often having only seconds to locate and process relevant evidence [7]. UpToDate is a widely utilized, evidence-based clinical decision support tool recognized for its expert-authored, regularly updated content and graded recommendations, making it a gold standard reference in many healthcare settings worldwide [8].

As healthcare increasingly incorporates technology, large language models (LLMs) such as ChatGPT (OpenAI, San Francisco, CA, USA) are emerging as potential tools within shared decision-making frameworks [9]. The rapid development of artificial intelligence (AI) has produced advanced LLMs whose potential utility in scientific and medical tasks has been explored in recent literature [10,11]. These tools offer benefits such as quick access to concise medical information. However, beyond readability, issues such as accuracy, citation transparency, and lack of clinician oversight remain important considerations for clinical applicability [12].

This study examines and compares content on AF management produced by ChatGPT-3.5 and UpToDate. The primary objective is to evaluate the readability of each source using standardized metrics such as the Flesch-Kincaid Grade Level (FKGL) [13] and Flesch-Kincaid Reading Ease (FRE) scores [12], to determine the appropriateness of AI-generated material for medical professionals and its potential role as a complementary tool to established clinical references.

Aims and objectives

To evaluate the readability of educational content on the management of AF produced by ChatGPT-3.5 compared with standard reference material from UpToDate, for use by medical professionals.

## Materials and methods

This cross-sectional original research study was conducted over one week, from June 21 to June 27, 2025. As the study did not involve human participants, identifiable data, or interventions, Institutional Ethics Committee approval was not required.

The topic selected for analysis was the management of AF. Educational content aimed at medical professionals was generated using ChatGPT-3.5 (accessed June 21, 2025), an LLM increasingly used in scientific and medical domains. The AI tool was prompted with the following four topics: (1) “Write an educational guide for medical professionals on control of ventricular rate in patients with atrial fibrillation," (2) "Write an educational guide for medical professionals on antiarrhythmic drugs to maintain sinus rhythm in patients with atrial fibrillation," (3) "Write an educational guide for medical professionals on management of atrial fibrillation: rate control vs. rhythm control," (4) "Write an educational guide for medical professionals on catheter ablation in atrial fibrillation.” The output was copied into a Microsoft Word document (Microsoft® Corp., Redmond, WA, USA) for evaluation.

For comparison, standard content on AF management was retrieved from UpToDate (accessed June 21, 2025), a widely used, evidence-based clinical decision support tool devised for healthcare professionals, which included detailed explanations and reviews of various topics in medicine with specific recommendations for treatment and diagnosis. Only the main text of the disease summary was used, excluding tables, references, and figure legends to maintain consistency.

Readability was assessed using the FRE score and FKGL via an online Flesch-Kincaid calculator [12]. Parameters evaluated included word count, sentence count, average words per sentence, FRE, FKGL, as well as the count and percentage of difficult words. These metrics allowed for a structured comparison of content accessibility for its intended audience - medical professionals.

Data were compiled in Microsoft Excel and analyzed using R software, version 4.3.2 (R Foundation for Statistical Computing, Vienna, Austria). The Mann-Whitney U test was used to compare readability scores between ChatGPT and UpToDate content. A p < 0.05 was considered statistically significant.

## Results

Educational content on AF for medical professionals was generated using ChatGPT and retrieved from UpToDate. The responses were evaluated for readability parameters, including word count, sentence count, average words per sentence, FRE, FKGL, the Simple Measure of Gobbledygook (SMOG) Index [14], difficult word count, and difficult word percentage.

FRE measures how easy a text is to read on a scale from 0 to 100. A higher FRE score indicates easier readability. For example, scores above 60 are easily understood by most people, while scores below 30 suggest college-level or technical content. FKGL indicates the U.S. school grade level needed to understand the text. A higher FKGL score reflects greater reading difficulty, meaning the text is suitable for more educated readers (e.g., FKGL 12 = 12th grade level). The SMOG Index estimates the years of education required to understand a piece of writing. A higher SMOG score means the text is more complex, often used for professional documents like legal or healthcare materials. A higher count of difficult words usually indicates lower readability, especially for general audiences or those with lower reading proficiency. Difficult word percentage is the proportion of difficult words compared to the total word count. A higher percentage signals a more challenging text, often requiring advanced vocabulary knowledge.

Table 1 compares the readability characteristics of ChatGPT and UpToDate content. The analysis was performed using IBM SPSS Statistics for Windows, Version 25 (Released 2017; IBM Corp., Armonk, New York, United States) and R software. The Mann-Whitney U test compared the distributions of readability measures generated by both sources. Based on the p-values obtained in Table 1, statistically significant differences were observed for median word count (p = 0.029), average words per sentence (p = 0.029), and difficult word percentage (p = 0.029).

**Table 1:** Comparison between readability characteristics between ChatGPT and UpToDate. ^+^ Mann-Whitney U test. * P-values <0.05 are considered statistically significant. FRE: Flesch-Kincaid Reading Ease; FKGL: Flesch-Kincaid Grade Level; SMOG: Simple Measure of Gobbledygook

	Median (IQR)		U Statistic	P-value^+^
	UpToDate	ChatGPT		
Word Count	3381.0 (1562.5-4046.0)	495.0 (198.2-845.0)	0	0.029*
Sentence Count	175.0 (83.5-195.2)	34.5 (13.2-86.5)	1	0.057
Word/Sentence Count	19.3 (18.3-20.7)	14.3 (10.3-15.3)	0	0.029*
FRE	32.5 (22.2-35.5)	24.4 (18.9-35.8)	6	0.686
FKGL	13.4 (13.1-15.2)	13.6 (11.6-14.3)	7.5	0.971
SMOG Index	12.1 (11.8-13.5)	12.0 (10.2-12.2)	6.5	0.743
Difficult Word Count	738.0 (364.8-1073.0)	151.5 (56.8-256.0)	1	0.057
Difficult Word Percentage	23.3 (21.6-27.0)	29.6 (28.5-31.0)	0	0.029*

**Figure 1:**
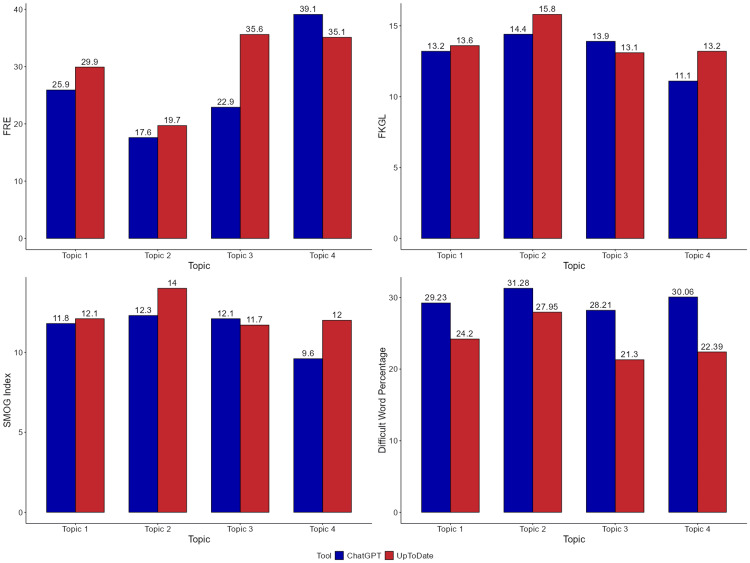
Graphical representation of comparison of readability metrics - Flesch Reading Ease (FRE), Flesch-Kincaid Grade Level (FKGL), SMOG Index, and difficult word percentage - for the patient education guide generated by UpToDate and ChatGPT. Topic 1: Write an educational guide for medical professionals on control of ventricular rate in patients with atrial fibrillation. Topic 2: Write an educational guide for medical professionals on antiarrhythmic drugs to maintain sinus rhythm in patients with atrial fibrillation. Topic 3: Write an educational guide for medical professionals on management of atrial fibrillation: rate control vs. rhythm control. Topic 4: Write an educational guide for medical professionals on catheter ablation in atrial fibrillation.

Figure 1 provides a graphical representation of the comparison of readability metrics - FRE, FKGL, SMOG Index, and difficult word percentage - for the patient education guide generated by UpToDate and ChatGPT. FRE scores were consistently higher for UpToDate, indicating greater ease of readability. The largest difference was observed in Topic 4 (ChatGPT: 39.1 vs. UpToDate: 35.1), although ChatGPT outperformed UpToDate in this topic. In Topics 1-3, UpToDate had higher scores, with the most notable difference in Topic 3 (ChatGPT: 22.9 vs. UpToDate: 35.6). FKGL and SMOG Index values showed minor variation between the two sources. In most topics, UpToDate had slightly higher grade levels, suggesting marginally more complex sentence structures. However, in Topic 3, the FKGL (13.9 vs. 13.1) and SMOG Index (12.1 vs. 11.7) were slightly higher for ChatGPT, indicating more advanced language use in that specific topic. Difficult word percentage was higher in content generated by ChatGPT across all topics, suggesting a greater presence of complex vocabulary. The largest gap was in Topic 4 (ChatGPT: 30.06% vs. UpToDate: 22.39%). These visual trends align with the statistical analysis, suggesting that while ChatGPT can produce clinically relevant educational material, it generally employs more complex language compared to UpToDate.

## Discussion

This cross-sectional study compared educational content generated by ChatGPT with UpToDate for medical professionals on AF. Table 1 revealed significant differences in word count, average words per sentence, and difficult word percentage (p = 0.029). ChatGPT generated shorter, denser text with a higher proportion of complex vocabulary. Figure 1 supported these findings, showing consistently lower FRE scores and higher difficult word percentages for ChatGPT across all topics.

AI tools are increasingly used to support clinical learning by generating fast, structured summaries. In this study, ChatGPT and UpToDate were compared for their ability to produce educational content on AF. ChatGPT generated shorter, more condensed responses with a higher proportion of difficult words, which may be useful for clinicians already familiar with the topic. However, lower FRE scores and a higher difficult word percentage suggest reduced readability, indicating a need for clearer structuring to enhance accessibility, particularly for early-career professionals or those new to the subject matter [12-18].

Readability scores such as FRE, FKGL, and SMOG Index assess how easily medical text can be understood. Higher FRE scores indicate greater readability. Studies have shown that AI-generated medical content often exceeds recommended reading levels, potentially limiting accessibility [19,20]. In this study, ChatGPT had lower readability than UpToDate, suggesting harder comprehension.

The findings align with prior evaluations of LLMs in medical education. For instance, Salvagno et al. (2023) found that ChatGPT-generated explanations for clinical topics were factually accurate but linguistically dense, requiring simplification for broader educational use [11,21]. Pillai and Pillai (2024) observed that AI-generated cardiology content was structurally well-organized but occasionally too technical for time-pressed learners [22]. Similarly, Kung et al. (2023) reported that ChatGPT passed the United States Medical Licensing Examination (USMLE) but often used verbose or overly formal phrasing, which could hinder rapid understanding in clinical settings [23]. These studies point to a consistent trend: AI tools can produce reliable and coherent summaries, but their readability often falls short of optimal, particularly in high-pressure environments.

However, this study’s findings diverge from earlier studies describing AI-generated text as overly simplistic or lacking depth. For example, Bubeck et al. (2023) found that earlier versions of LLMs produced generic, surface-level answers that failed to capture clinical nuance [24]. In contrast, this study showed that ChatGPT generated dense and terminology-rich responses with lower FRE scores and higher difficult word percentages than UpToDate, suggesting more complex, rather than simplified language. These differences may be explained by updates in model architecture, the clinical prompt used, or variation in evaluation methods. The evolving capabilities of LLMs likely contribute to differing outcomes across studies, emphasizing the importance of context when assessing educational usefulness.

Limitations

This study has several limitations. It focused on a single AI tool (ChatGPT) and one clinical topic (AF), limiting the generalizability of findings. The version of ChatGPT used may not reflect the most up-to-date clinical knowledge, as it was not benchmarked against evolving guidelines. The version of ChatGPT used for the study is not available or accessible anymore. Furthermore, while readability metrics provided quantitative insight, this study did not assess the clinical accuracy or practical utility of the content through expert review. Another limitation of this study is the exclusive use of the Mann-Whitney U test for statistical analysis. The absence of multivariate analysis and effect size reporting limits the depth of interpretation, making it difficult to assess the strength and practical significance of the observed differences between groups. Future research should explore multiple AI platforms across diverse specialties, incorporating input from educators and clinicians to fully evaluate the role of generative AI in medical education.

## Conclusions

This study compared educational content on AF generated by ChatGPT with that from UpToDate, focusing on readability for medical professionals. Key differences were identified in readability scores and language complexity, with ChatGPT producing more concise but denser and less readable content. While both sources offered clinically relevant information, the findings suggest that AI-generated materials may require refinement to enhance clarity and accessibility, particularly in time-sensitive clinical settings. AI tools like ChatGPT hold potential as supplementary resources in medical education, but their use must be accompanied by critical evaluation. Further research is needed to explore their performance across different medical topics, tools, and user needs.
